# Relationships Between Vitamin D, Gut Microbiome, and Systemic Autoimmunity

**DOI:** 10.3389/fimmu.2019.03141

**Published:** 2020-01-21

**Authors:** Erin A. Yamamoto, Trine N. Jørgensen

**Affiliations:** ^1^Cleveland Clinic Lerner College of Medicine of Case Western Reserve University, Cleveland, OH, United States; ^2^Department of Inflammation and Immunity, Lerner Research Institute, Cleveland Clinic, Cleveland, OH, United States

**Keywords:** microbiome, bacterial composition, gut barrier, vitamin D, autoimmune disease

## Abstract

There is increasing recognition of the role the microbiome plays in states of health and disease. Microbiome studies in systemic autoimmune diseases demonstrate unique microbial patterns in Inflammatory Bowel Disease, Rheumatoid Arthritis, and Systemic Lupus Erythematosus to a lesser extent, whereas there is no single bug or pattern that characterizes Multiple Sclerosis. Autoimmune diseases tend to share a predisposition for vitamin D deficiency, which alters the microbiome and integrity of the gut epithelial barrier. In this review, we summarize the influence of intestinal bacteria on the immune system, explore the microbial patterns that have emerged from studies on autoimmune diseases, and discuss how vitamin D deficiency may contribute to autoimmunity via its effects on the intestinal barrier function, microbiome composition, and/or direct effects on immune responses.

## Introduction

Nearly 15 million people in the United States are living with an autoimmune disease, and this number increases annually (1). In autoimmunity, the immune system recognizes, targets, and causes damage to normal tissues such as skin, kidney, pancreas, the nervous system, joints etc. Vitamin D deficiency has long been associated with systemic autoimmune disease and is suspected to play a role in disease pathogenesis.

Although vitamin D is well-known for its role in calcium homeostasis it also has numerous direct and indirect regulatory effects on the immune system that briefly include promoting regulatory T cells (Tregs), inhibiting differentiation of Th1 and Th17 cells, impairing development and function of B cells, and reducing monocyte activation [reviewed in (2, 3)]. Given its predominantly immunosuppressive effects, vitamin D could be of therapeutic benefit. In fact, many preclinical studies in multiple sclerosis (MS) and colitis models (fewer in arthritis and lupus) have demonstrated benefit to oral or intraperitoneal administration of vitamin D (3). However, unequivocal benefit has not been achieved in clinical studies, suggesting that the relationship between vitamin D and autoimmunity is more complicated than originally believed. It remains unclear whether vitamin D can act through mechanisms alternative to immunosuppression to impact autoimmunity.

The human microbiome is “the ecological community of commensal, symbiotic, and pathogenic microorganisms” that survive on/in our bodies (4). It consists of 12 different bacterial phyla, with 93.5% classified as Bacteroidetes, Proteobacteria, Firmicutes, Actinobacteria, or Euryarchaeota phyla (5, 6). Intestinal microbes help us digest foods into compounds and nutrients that can be absorbed and utilized by the body. In the last 10 years, it has become evident that the gut microbiome plays an important role in shaping the immune system, and contributing to health and disease (7–9). The microbiome is of particular interest in autoimmunity due to “molecular mimicry,” the concept that foreign microbial peptides might share structure and sequence similarities with self-antigens and are thus capable of initiating immune cell auto-reactivity.

In this review we explore the interaction between microbiome and autoimmunity and the ways in which vitamin D might influence this interaction to facilitate autoimmune disease.

## Gut Microbiome Influences Immune Responses and Autoimmune Disease

Evidence of dysbiosis, alterations in gut flora composition, in autoimmunity is becoming increasingly concrete. However, exactly how the microbiota and the immune system interact directly or indirectly to promote disease remains unknown. Despite variation in dysbiosis across autoimmune diseases, there is evidence to suggest that specific bacteria differentially promote or inhibit immune responses, collectively implying that there may be a greater polymicrobial influence on inflammatory states.

**Figure 1:**
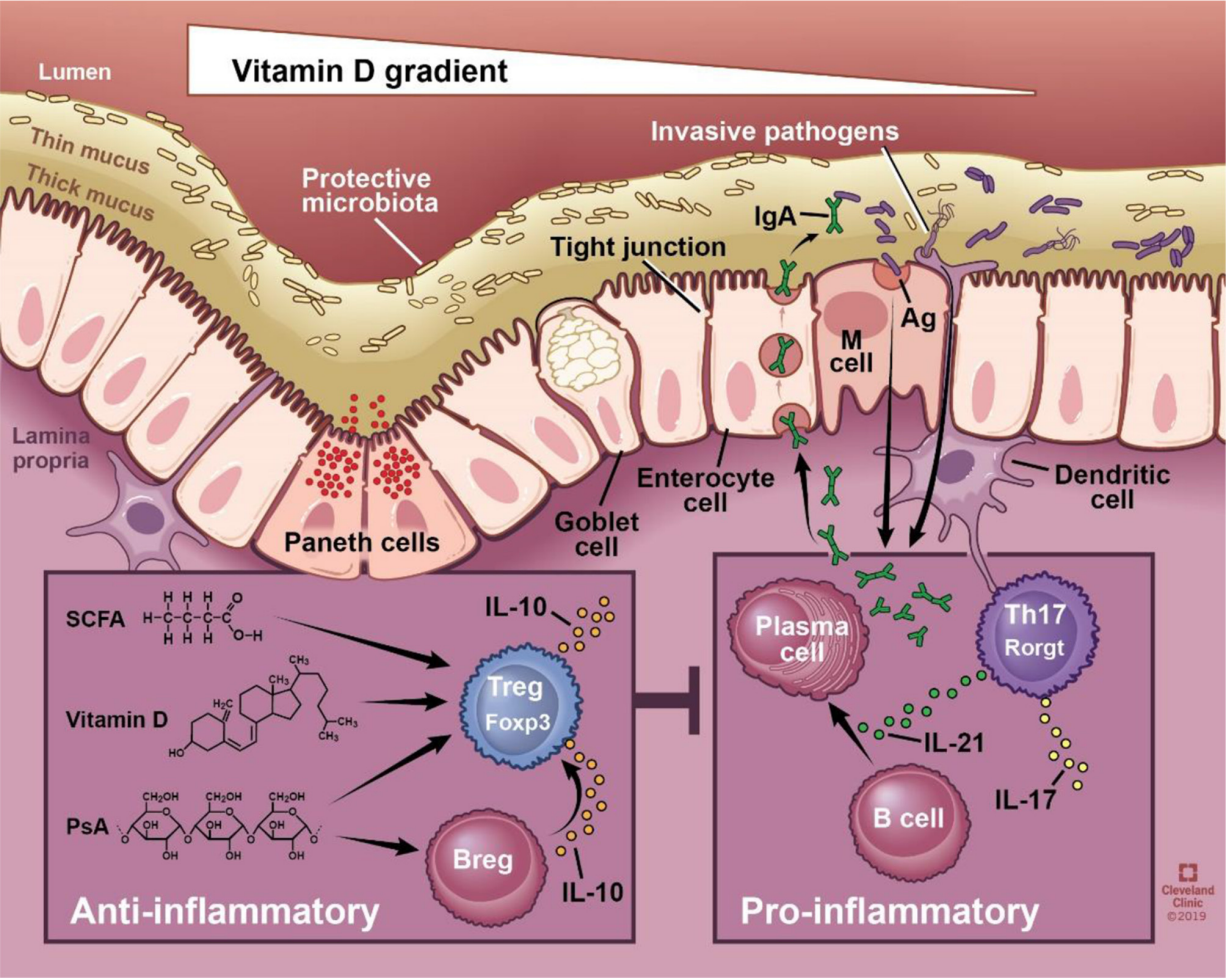
Schematic of the physical and functional intestinal epithelial barrier. The physical barrier is composed of a thin and thick mucus layer, followed by single cell layer consisting of enterocytes, Paneth cells, goblet cells, and microfold (M) cells. The integrity of this layer is maintained via intact tight junctions. Functionally, the epithelium produces mucin and antimicrobial peptides, and allows translocation of secretory immunoglobulin A. Intestinal immune cells sample the luminal environment, respond to invasive pathogens, and coordinate innate and adaptive immune responses. SCFA, vitamin D, and polysaccharide A have all been shown to promote regulatory adaptive responses, whereas bacteria generally promote proinflammatory responses. SCFA, short chain fatty acids; PsA, polysaccharide A; sIgA, secretory IgA; Ag, antigen; M cell, microfold cell. Illustration by David Schumick, BS, CMI. Reprinted with the permission of the Cleveland Clinic Center for Medical Art & Photography © 2019. All rights reserved.

The gut barrier is a physical and functional barrier between host cells and the external environment composed of outer and inner mucus layers, intestinal epithelial cells, immune cells of the lamina propria, and gut-associated lymphoid tissues (GALT). A mucus layer, produced by goblet cells, physically prevents bacteria from coming into contact with the host. If the mucus layer is breached, a single layer of intestinal epithelial cells acts as the next line of defense. This layer is composed of specialized epithelial cells including enterocytes, Paneth cells, goblet cells, and microfold cells each providing a unique mechanism of protection ranging from phagocytosis to secretion of antimicrobial peptides and IgA [reviewed by (10)] (Figure 1). The proposed function of secretory IgA produced by plasma cells ranges widely to include binding bacteria to prevent host interaction, promoting downregulation of inflammatory epitopes or toxin neutralization, and coating bacteria for subsequent interaction with the host immune system in peyer's patches [reviewed in (11)]. The intestinal epithelial cell layer maintains its impermeability to pathogens and toxins via intact tight junctions. Disruption to any component of the physical or functional gut barrier raises host susceptibility to pathogen invasion and subsequent interaction with the host immune system.

### Intestinal Bacteria Influence Inflammation and Immune Responses

Intestinal immune cells are primarily found within the lamina propria and GALT. GALTs are comprised of B cell rich lymphoid follicles reminiscent of lymph nodes, and are associated with overlying specialized follicle-associated epithelium containing M cells that facilitate antigen entry from the intestinal lumen. In some regions of the gut, dendritic cells extend their dendrites through the epithelial layer to sample antigen from the lumen (12). Through these mechanisms, resident dendritic cells and T cells acquire access to luminal antigens and promote B cell differentiation and class switch recombination into IgA-producing cells. Plasmablasts home to the intestinal lamina propria where they differentiate into plasma cells (13).

The resident bacterial community is critical for proper immune function. It has been shown that antibiotic mediated depletion of gut microbiota disrupts this relationship and impairs normal innate immune responses such as type I and type II IFN responses by macrophages (14). The presence of bacteria within intestinal macrophages specifically induces IL-1β without an effect on IL-6, which drives differentiation of Th17 cells (15).

Commensal bacteria coexist within the intestines mediating effects on the immune system in a balancing act that maintains homeostasis [reviewed by (16)]. Whereas, *Bacteroides fragilis* has an inhibitory effect on Th17 cells, segmented filamentous bacteria (SFB) have a well-documented ability to promote a Th17 response (17–19). This response is dependent on SFB adherence to intestinal epithelial cells (20) via cell wall glycopolymers which are common to gram-positive bacteria (21); however adherent gram-negative bacteria are also capable of inducing Th17 responses (20). Furthermore, coinfection with SFB and *Listeria monocytogenes* generated Th17 and Th1 cells, respectively, demonstrating an important concept that individual bacteria can elicit specific immune cell responses (22). Colonic Tregs are also capable of undergoing expansion in response to certain bacterial species. For example, a cocktail of Clostridial strains isolated from healthy human fecal samples reduced features of TNBS-associated colitis and allergic diarrhea models via Treg upregulation (23, 24).

In the intestines, B cells primarily localize to the lamina propria (LP). LP B cells were found to express *Rag2* and DNA polymerase, characteristics of pro-B cells, which suggests that B cell development may occur in the gut (25). Interestingly, colonized germ free mice (by weaning with specific pathogen free mice for 7 days) had significantly increased *Rag1* and *Rag2*, and increased percentages of pro-B cells (CD19+ B220-low CD43+) in the bone marrow, spleen, and LP compared to their germ free littermates (25). Furthermore, germ free mice display reduced numbers of IgA+ plasma cells that increased in response to colonization [(26, 27), and reviewed in (13)]. Thus, gut microbiota is associated with and may potentially serve as an antigen source for immature B cell development in the gut.

The interaction between microbiota and B cells also influence one another to maintain homeostasis. In arthritis-induced mice, gut colonization stimulated IL-1β and IL-6 production which promote the development and function of splenic and mesenteric lymph node IL-10-producing B cells (28). Furthermore, colonic bacterial lysate or specific species such as *B. fragilis*, have also been shown to stimulate IL-10-producing B cells, which are capable of suppressing T cell mediated inflammation and colitis (29–31). These interactions, by promoting immunoregulatory activity, contribute to our ability to live symbiotically with bacteria.

The Bacteroidetes phylum is the largest phylum of gram-negative bacteria and has a reputation of promoting health. Within this phylum, the *Bacteroides* genus is the most prevalent in the gut (32). Polysaccharide A (PsA), a component of the *B. fragilis* cell wall, has been widely studied. PsA induces IL-10 production by intestinal T cells, possibly via ligation of TLR2 on plasmacytoid dendritic cells (33). Induction of regulatory T cells (Treg) was shown to be dependent on IL-10-producing B cells, and protected against herpes encephalitis (29). Corresponding with this immunoregulatory response, PsA inhibits Th17 cell expansion, while a modified *B. fragilis* that lacks PsA loses the ability to induce IL-10 production and becomes proinflammatory (34, 35).

As discussed, the literature supports that microbiota promote both humoral immunity (B cell development and proinflammatory T cell responses) as well as immune regulation (regulatory B and T cells). In MS, multiple studies have shown that disease improves with B cell depletion (rituximab, anti-CD20), yet is exacerbated by neutralization of a B cell growth factor (atacicept, TACI-Ig) [reviewed in (36)]. It can be inferred from these data that there are disease-promoting and disease-fighting B cell subsets, and that it is possible that these specific cell subsets are differentially influenced by microbial influences.

Metabolites produced by intestinal bacteria (e.g., short chain fatty acids, lipids, vitamins) also play an important role in immune modulation [reviewed by (37, 38)]. Short chain fatty acids (SCFA; e.g., butyrate, acetate, propionate) are byproducts of dietary fiber fermentation in the large intestine. In general, the Bacteroidetes phylum primarily produce acetate and propionate, whereas the Firmicutes phylum mainly produce butyrate (39), though this is a simplification. Butyrate and propionate, but not acetate, were shown to promote extrathymic Treg differentiation (40, 41). Additionally, butyrate leads to a downregulation of LPS-induced proinflammatory cytokine production (i.e., NO, IL-6, IL-12) by intestinal macrophages (42), further supporting butyrate as an anti-inflammatory metabolite. There is also increasing data suggesting that SCFAs help maintain blood-brain barrier integrity (43) which is believed to contribute to neurologic conditions including MS that are being increasingly associated with the gut [reviewed in (38)]. Secondary bile acids (i.e., deoxycholic acid and lithocholic acid) are converted from primary bile acids by colonic bacteria. Activation of bile acid activated receptors by secondary bile acids triggers an anti-inflammatory response characterized by increases in *Tgfb, Il10*, and *Foxp3* gene expression, and suppression of NF-kB mediated expression of proinflammatory cytokines (*Il6, Tnfa, Il1b*, and *Ifng*) (44, 45). Finally, a variety of metabolites including secondary bile acids, fatty acids, and secondary metabolites, act intracellularly to regulate transcription or act on metabolite sensing-G protein-coupled receptors to regulate inflammatory leukocytes, Tregs, and/or modulate the intestinal barrier [reviewed by (46)].

## Microbial Dysbiosis and Autoimmunity

The intestinal microbiome, its relative composition, as well as alterations to that composition are capable of modifying autoimmune severity. The effect of the microbiome has been well-studied primarily by means of bacterial depletion (germ-free models or antibiotics), enrichment of single or several specific bacterial species, or complete fecal microbiota transplant.

Oral antibiotic treatment depletes bacteria residing in the intestinal tract allowing for assessment of disease in the absence of bacterial colonization. In the MRL mouse model of Systemic Lupus Erythematosus (SLE), oral antibiotic treatment improved nephritis (47), and in the experimental autoimmune encephalitis (EAE) model of MS, oral, but not intraperitoneal antibiotics, impaired the development of EAE (48). Similarly, germ-free animal models of MS (49), SLE (50), IBD (51), and RA (collagen-induced arthritis) (52) have shown attenuated autoimmunity compared to conventionally housed animals. Interestingly, adjuvant-induced arthritis under germ free conditions has been shown to induce a more severe disease when compared to conventional adjuvant-induced arthritis (53, 54). Whether this is due to an abnormal response to adjuvants in these mice or reflective of different autoimmune processes is not clear. It should also be noted that mice bred under gnotobiotic conditions are known to have underdeveloped immune systems, and results from these studies should be interpreted with caution (34). Despite the positive effects of antibiotic treatment, it remains unclear whether disease improvement occurs as a result of reinstating a healthier balance of “good” and “bad” microbes, by replenishing microbial populations that provide metabolites beneficial to the host, or depletion of proinflammatory bacteria.

Clinical studies are underway to determine what differences exist, if any, between the gut microbiota of autoimmune patients and healthy controls, how current therapies or diet affect microbiome composition, and whether fecal transplant can normalize microbiome composition and ameliorate disease features (NCT03183869, NCT03926286, NCT03797937, NCT02580435, NCT01619176, NCT03775824, NCT02544334, NCT02736279, NCT03423121, NCT03802890, NCT03594487, NCT01198509). There are more data generated from the completed studies in IBD and MS compared to the few studies performed in rheumatic diseases, which are now gaining traction. Here we highlight selected microbial differences from these studies.

### Multiple Sclerosis

When comparing taxonomic differences between relapsing-remitting MS and healthy controls in Japan, the abundance of 21 species significantly differed between the groups. Of these, 14 of 21 were clostridial species (cluster XIVa, IV), and all were reduced in MS samples (55). Convincingly, significant differences in the 21 species were sustained in a follow-up longitudinal fecal microbial analysis (55). Additionally, fecal samples showed significantly lower representation of *Bacteroides* and *Prevotella* (phylum: Bacteroidetes), *Faecalibacterium* and *Anaerostipes* (phylum: Firmicutes), and an increased representation of *Bifidobacterium* (phylum: Actinobacteria) and *Streptococcus* (phylum: Firmicutes) genera in MS patients with and without ongoing therapy (Table 1).

**Table 1:** Overview of identified bacteria of significant enrichment or depletion in autoimmune human gut microbiome studies compared to healthy gut microbiota.

	**Multiple sclerosis**	**IBD**	**Rheumatoid arthritis**	**Lupus**
**Enrichment**	Actinobacteria Bifidobacterium (55) Bacteroidetes *Pedobacter* (56) Euryarchaeota *Methanobrevibacter* (57) Firmicutes *Megasphaera* (57) *Blautia* (56) *Dorea* (56) *Streptococcus* (55) Proteobacteria *Pseudomonas* (56) *Mycoplana* (56) *Acinetobacter* (58) **Verrucomicrobia** ***Akkermansia*** **(****57****–****59****)**	**Fusobacterium (****60****–****62****)** **Proteobacteria** ***Escherichia coli*** **(****63****–****65****)**	Actinobacteria *Eggerthella* (66) *Eggerthella lenta* (67) *Gordonibacteria pamelaeae* (67) **Bacteroidetes** ***Prevotella copri*** **(****68****–****71****)** Firmicutes *Clostridium asparagiforme* (67) *Lachnospiraceae bacterium* (67) *Lactobacillus salivarus* (67)	Actinobacteria *Rhodococcus* (72) *Eggerthella* (72) Bacteroidetes *Prevotella* (72) *Bacteroides* (73) *Alistipes* (73) *Barnesiella* (73) Firmicutes *Lachnoclostridium* (73) *Lachnospira* (73) Proteobacteria *Klebsiella* (72)
**Depletion**	Actinobacteria *Adlercreutzia* (56) *Collinsella* (56) Bacteroidetes *Bacteroides* (57) *Prevotella* (55, 57) *Butyricimonas* (57) *Paraprevotella* (56) ***Parabacteroides*** **(****56**, **58****)** Firmicutes *Anaerostipes* (55) *Faecalibacterium* (55) *Lactobacillus* (56) Proteobacteria *Sutterella* (56)	**Firmicutes** ***Faecalibacterium prausnitzii*** **(****64**, **65**, **74**, **75****)** Bacteroidetes *Bacteroides* (76)	Actinobacteria *Bifidobacterium bifidum* (67) Bacteroidetes *Bacteroides* spp. (67) Firmicutes *Ruminococcus lactaris* (67) Proteobacteria *Haemophilus* spp. (67) *Klebsiella pneumoniae* (67)	Firmicutes *Pseudobutyrivibrio* (72) *Dialister* (72) *Clostridium sensu stricto1* (73) *ErysipelotrichaceaeUCG* (73) *ChristensenellaceaeR* (73) *Romboutsia* (73) **Firmicutes:Bacteroidetes ratio** **(****72**, **73**, **77**, **78****)**

*The taxonomic level shown was determined by each study under the respective phylum. Bold-type indicates that the phylum or species was cited by multiple sources*.

In the Chinese population, MS patient (treated and untreated) microbiota were enriched with *Pseudomonas and Mycoplana* (phylum: Proteobacteria), *Blautia* and *Dorea* (phylum: Firmicutes), and *Pedobacter* (phylum: Bacteroidetes); whereas healthy controls showed enrichment with *Adlercreutzia* and *Collinsella* (phylum: Actinobacteria), *Lactobacillus* (phylum: Firmicutes) and *Parabacteroides* (phylum: Bacteriodetes) (56) (Table 1).

In contrast, an American study reported enrichment of *Methanobrevibacter* (phylum: Euryarchaeota), *Akkermansia* (phylum: Verrucomicrobia), and *Megasphaera* (phylum: Firmicutes), and a reductions of *Butyricimonas* (phylum: Bacteroidetes), *Paraprevotella* (phylum: Bacteroidetes), *Slackia* (phylum: Actinobacteria), *Collinsella* (phylum: Actinobacteria), *Prevotella* (phylum: Bacteroidetes), and *Sutterella* (phylum: Proteobacteria) in untreated MS patients compared to healthy controls (57) (Table 1). In this study it was shown that *Methanobrevibacter* and *Akkermansia* were associated with the expression of proinflammatory genes, while *Butyricimonas* associated with anti-inflammatory gene expression changes in T cells, dendritic cells, and monocytes (57). Interestingly, MS patients in remission were more similar to healthy controls than to active MS patient microbiota in terms of species richness and overall diversity (56), suggesting that active disease could be associated with gut microbiome composition. Furthermore, there is also evidence to suggest that MS treatment modifies bacterial communities (79).

Another American study found *Acinetobacter* (phylum: Proteobacteria) and *Akkermansia* (phylum: Verrucomicrobia) to be enriched, and *Parabacteroides* (phylum: Bacteroidetes) was reduced in MS stool samples compared to healthy controls (58). Bacterial species from these genera, *Acinetobacter calcoaceticus* and *Parabacteroides distasoni*, inhibited FoxP3+ Treg differentiation and stimulated CD4+IL-10+ lymphocytes, respectively, and *Akkermansia muciniphila* promoted differentiation of IFNg+ Th1 lymphocytes *in vitro*. Together, these functional effects demonstrate that unique MS-associated bacteria might affect different immune cells to promote inflammation.

In a fascinating European twin study, there were no observed overall differences in gut bacterial taxonomy (59). However, in subgroup analysis of 15 untreated pairs of twins, there was significant enrichment of *A. muciniphila*, although this finding appears to primarily be driven by 3 twin pairs. In both the Cekanaviciute and Berer studies, MS patient fecal transplant to EAE mice elicited more severe EAE symptoms than fecal transplant from healthy controls/twins (58, 59).

These studies highlight the heterogeneity that exists between studies in the MS literature and the difficulty of performing cross-cultural comparisons with regard to microbiome composition across patient populations. Differences in dietary intake, environmental exposure, disease activity and treatment status likely all contribute to these microbial differences. While disease activity and treatment status can be easily followed and accounted for, diet and environmental influences will remain as uncontrolled and confounding variables.

### Inflammatory Bowel Disease

Over the last decade, subtle patterns have emerged in the IBD microbiome (80) that appear to span cross-cultural differences. There is a general reduction in species richness or alpha diversity in the fecal microbiome (81) that aligns with changes in mucosal areas of inflammation (82). Additionally, adherent-invasive bacteria including *Escherichia coli* (phylum: Proteobacteria) and *Fusobacterium* (phylum: Fusobacteria) appear to be enriched in IBD patients from North America, Japan, Italy (60–62, 83, 84) (Table 1). Enrichment of *E. Coli* may also contribute to the observed increase in the *Gammaproteobacteria* class in IBD (63).

Reciprocally, there are also bacteria found to be reduced in IBD that promote intestinal health. *Faecalibacterium prausnitzii* (phylum: Firmicutes), one of the most prevalent gut bacterial species, is reduced in feces of ulcerative colitis (UC) patients (64, 74, 85) and feces/mucosal biopsies from Crohn's disease (CD) patients (65, 86) (Table 1), corresponding with reported reductions in the Firmicutes phylum (63, 87). Interestingly, *F. prausnitzii* promotes gut health through its role as a primary producer of butyrate and its ability to promote a tolerogenic/anti-inflammatory cytokine profile (74, 85, 86, 88). A meta-analysis of 9 studies found the *Bacteroides* genus to be reduced in UC and CD patients compared to healthy controls (76) (Table 1). *Bacteroides thetaiotaomicron* is an acetate producer that promotes goblet cell differentiation and mucus production, ultimately providing protection against intestinal inflammation by strengthening the gut barrier (89). Recently, additional studies suggested *B. thetaiotaomicron* colonization demonstrated therapeutic properties in preclinical models of CD (90), although separate studies showed that *B. thetaiotaomicron* can be pathogenic in patients with certain genetic susceptibilities (75, 91). Finally, alterations to bacterial colonization can lead to downstream metabolic or indirect effects on other bacteria. For example, large amounts of acetate produced by *Bifidobacterium longum* is capable of preventing translocation of toxins across the intestinal epithelium in mice infected with *E. coli* O157 (92), which suggests that at least some bacteria have indirect effects on other bacterial species.

Evidence from animal models suggests that fecal transplant, which has been successfully used to treat *Clostridium difficile* infection, would have therapeutic benefit in IBD. As such, fecal microbiota transplant from normobiotic mice to mice with DSS-induced resulted in reduced intestinal inflammation as evidenced by improvement in colon length and colon histological scoring (reductions in inflammatory cell infiltrate, extent, hyperplasia, glandular rarefaction, dysplasia, and granulation tissue) and increased anti-inflammatory antimicrobial peptides including *Camp* and *S100a8*, as well as upregulation of cell surface mucin expression, *Muc1* and *Muc4* (93, 94). Unfortunately, individual clinical studies have shown variable success in demonstrating benefit from fecal transplant, and two (95, 96) of four (97, 98) randomized controlled studies were stopped early for futility at interim analysis. Still, multiple meta-analyses and systematic reviews have concluded that fecal transplant is likely beneficial for at least short-term improvement of clinical symptoms (98–100). In summary, there are many aspects to this treatment that need to be defined such as possible long-term benefits or adverse effects, the best route and frequency of fecal transplant, how to select donors, and whether donor samples should be pooled prior to administration as it appears there are some donors whose fecal sample appears to provide greater benefit than others (95, 101).

### Rheumatoid Arthritis

An interaction between the gut and arthritis had been suspected decades prior to the recent expansion of interest in the microbiome (53, 54, 102). A recent animal study demonstrated that arthritis susceptibility can be transferred to germ-free mice conventionalized with fecal microbiota isolated from mice susceptible to collagen-induced arthritis, resulting in increased frequency of arthritis induction and exacerbated arthritic changes compared to conventionalization with microbiota from non-arthritis susceptible mice (52). Multiple studies have now demonstrated dysbiosis in RA patients (67–69, 103).

By 16S sequencing, *Prevotella copri* appears to be consistently enriched in new/early onset, treatment-naïve RA patients, as well as in first degree relatives of RA patients who did not meet RA diagnostic criteria (68–70). Additionally, a specific peptide sequence from *P. copri* (Pc-p27), was enriched in RA. Pc-p27 is capable of stimulating Th1 responses in new-onset RA, and generating IgG and IgA responses to *Prevotella* (71). Although these findings do not confirm pathogenicity of *P. Copri*, IgG antibody responses may be beneficial in facilitating RA diagnosis, though further studies are needed. In contrast, a study by Chen et al. did not identify *P. copri* in RA patients, but found *Eggerthella* (phylum: Actinobacteria) to be abundant in RA (66) (Table 1). The studies that identified *P. copri* focused on early, untreated RA patients or pre-clinical RA first degree relatives, whereas Chen et al. compared chronic, treated RA patient microbiome against controls. This suggests there may be differences between pre- and post-treatment RA groups.

Metagenomic shotgun sequencing has allowed the investigation of the RA microbiome based on numerous microbial genetic markers (67). Metagenomic linkage groups (MLG) enriched with treatment-naïve RA patients included *Clostridium asparagiforme* (phylum: Firmicutes)*, Lachnospiraceae bacterium* (phylum: Firmicutes)*, Gordonibacteria pamelaeae* (phylum: Actinobacteria), and *Eggerthella lenta* (phylum: Actinobacteria), in RA fecal microbiome compared to healthy controls (67). In contrast, MLGs containing *Klebsiella pneumoniae, Bacteroides* spp*., Bifidobacterium bifidum*, and *Ruminococcus lactaris* negatively correlated with RA MLGs. In addition, although MLGs were found to differ between dental plaque, saliva, and feces, *Haemophilus* spp. were depleted and *Lactobaillus salivarius* was overrepresented at all sites (Table 1). This group further found that salivary MLGs correlated with clinical indicators of RA such as disease activity score, anti-CCP, IgG, and CRP (67). Interestingly, *P. Copri* was not identified in this study using metagenomics sequencing, highlighting potential discrepancies that may exist between sequencing approaches.

### Systemic Lupus Erythematosus (SLE)

Relatively few investigations have studied the lupus microbiome in depth. As a result there is little consensus regarding patterns of dysbiosis in lupus. Current data focuses on the relative abundance of Firmicutes and Bacteroidetes with four independent studies (from Spain, China, Netherlands) reporting a decreased Firmicutes-to-Bacteroidetes (F:B) ratio compared to healthy individuals (72, 73, 77, 78). Although findings from a fifth study did not support a reduction in the F:B ratio (104), the four studies crossed cultural, dietary, and geographic influences which adds to the robustness of data (Table 1).

In the Chinese population, SLE patients had multiple significantly increased genera including *Prevotella* (phylum: Bacteroidetes), *Rhodococcus* and *Eggerthella* (phylum: Actinobacteria) and *Klebsiella* (phylum: Proteobacteria), with decreased abundance of Firmicutes driven primarily by reductions in *Pseudobutyrivibrio* and *Dialister* genera (72). A recent study found the SLE microbiome has 7 genera of increased abundance and 19 genera of decreased abundance compared to the general population (73) (Table 1), but these 26 genera had no overlap with the Chinese study despite both studies reporting a reduction in the F:B ratio. Thus, the two study populations seem to achieve this result via different bacterial genera.

Lupus prone SNF1 mice treated with acidic pH water had higher levels of intestinal Firmicutes, a reduction in Th17 activity, and delayed onset of nephritis compared to neutral pH water (105). Additionally, in the MRL model of SLE, increased abundance of *Lactobacillales* (phylum: Firmicutes) in the gut promoted mouse survival and improved renal pathology (47). As mentioned above, SLE patients has been characterized to have reduced Firmicutes compared to Bacteroidetes (72, 73, 77, 78), supporting a possible therapeutic role for the Firmicutes phylum in lupus. However, a robust study highlighted *Enterococcus gallinarum*, a gram-positive gut commensal bacterium of the Firmicutes, as a lupus-like disease promoting bug (106). *E. gallinarum* was isolated from the mesenteric vein, lymph node, and liver of (NZWxBXSB)F1 hybrid mice, and induced autoantigens (ERV gp70 and B2GPI), as well as type I IFN and proinflammatory cytokines. Finally, vaccination using heat-killed *E. gallinarum* reduced serum autoantibody levels and improved survival (106). While there seems to be increasing support of an F:B ratio in lupus, further work is needed to determine whether the F:B ratio is a cause or consequence of disease, and how the F:B ratio might affect the pathogenicity of other bacteria such as *E. gallinarum*.

### Treatment-Induced Alterations

Multiple studies suggest that disease modifying treatment affects the gut microbiome. In RA, only the studies of untreated patients identified *P. copri* as a notable RA-associated bacteria (66), and RA microbiota composition was shown to change following treatment with disease modifying anti-rheumatic drugs toward a composition more representative of a “healthy” microbiome phenotype (67). In an MS study on twins, significant differences in bacterial composition were only detected between twins when the MS-twin was untreated (59). These few studies support that treatment directly or indirectly alters the microbiome. Despite the observation that dysbiosis associates with autoimmunity and also normalizes with treatment, it remains unclear whether dysbiosis or inflammation is the precipitating factor. This “chicken or the egg” phenomenon presents a particularly frustrating dilemma for the field.

This overview of the acquired gut microbiome data are only highlights of the vast changes that can be observed, representing another challenge in studying the microbiome. In addition, the findings support that unique alterations to the microbiome are likely associated with different autoimmune diseases. Interestingly, while some data support bacterial mediated immune regulation of disease (e.g., *F. Prausnitzii* in colitis), there are also microbial patterns that support a role for molecular mimicry (e.g., *P. Copri* in RA) suggesting the involvement of multiple mechanisms. Data from Neuromyelitis Optica showed an association with *Clostridium perfringes* as well as sequence homology between *C. perfringes* protein and self-antigen aquaporin-4 in the central nervous system [reviewed in (107)]. Such clear associations have yet to be observed in systemic autoimmunity. If molecular mimicry plays a significant role, it likely requires multiple foreign antigens with shared homology given the diversity of tissues affected in systemic autoimmune disease. This might involve multiple antigens from a single microbe or multiple antigens from many microbes. Furthermore, in order for molecular mimicry to occur, there must be exposure to the microbe, either by a proliferative advantage or host susceptibility, the details of which must also be understood.

## Vitamin D and Immune Defense in the Gut

Vitamin D is well-known for its role in calcium homeostasis and bone growth, but is also well-studied for its anti-inflammatory properties [reviewed by (2, 3)]. Briefly, vitamin D classically acts through the vitamin D receptor (VDR) to regulate gene transcription. Within the immune system, vitamin D inhibits Th17 and Th1 responses, promotes Tregs, impairs B cell development and function, and stimulates antimicrobial peptides from immune cells. In this section we will focus on how vitamin D specifically affects microbiota composition and the gut barrier.

### Influence of Vitamin D on Gut Microbiome Composition

It has recently been shown that the composition of the gut microbiome can be altered by vitamin D status/exposure (108, 109). Rodent studies demonstrate that vitamin D deficiency by dietary restriction, lack of CYP27B1, or lack of VDR promote increases in the Bacteriodetes (109–112) and Proteobacteria phyla (109, 110, 112). Furthermore, a recent GWAS identified two VDR polymorphisms as significant contributors to microbiota variation within a combined cohort of 2029 individuals from the general German population and patients with specific disease entities (e.g., autoimmune disease, metabolic syndrome, sarcoidosis) (113). In this study, human VDR polymorphisms consistently influenced the genus *Parabacterioides* (phylum: Bacterioidetes), and subsequent evaluation of VDR^−/−^ mice showed a corresponding increased abundance of *Parabacteroides* compared to WT mice (113).

Human studies have reported significant associations between vitamin D and microbiome composition. In a cross-sectional study of healthy individuals, vitamin D intake was negatively associated with abundance of *Prevotella* and strongly positively associated with *Bacteroides*, both of the phylum Bacteroidetes (114). In contrast, Luthold et al. found that healthy individuals with higher reported vitamin D intake had greater fecal abundance of *Prevotella*, and reduced *Haemophilus* (phylum: Proteobacteria) and *Veillonella* (phylum: Firmicutes) (108). In the same study, bacterial enrichment differed in individuals with higher serum 25(OH)D, as they displayed greater abundance of *Megaphaera* (phylum: Firmicutes), yet maintained a reduction in *Veillonella* and *Haemophilus* (108). In a study that utilized endoscopy and colonoscopy biopsies in addition to stool samples, it was found that 8-weeks of vitamin D3 supplementation resulted in increased species richness in the gastric antrum, decreased Proteobacteria (specifically *gammaproteobacteria*) in the upper GI tract (gastric corpus and gastric antrum), and increased Bacteroidetes (gastric corpus and descending duodenum) (115). Of note, microbial composition of the lower GI tract and stools did not differ between pre- and post-vitamin D3 treatment (115), suggesting that stool sample analysis may not be the appropriate means to study the effect of vitamin D3 on microbial communities. In support of this, an observational study did not find an association between habitual vitamin D intake and relative abundance of fecal bacterial genera (116). Whether vitamin D influences microbial composition along the GI tract vs. in stool is of particular importance and raises caution regarding the location of fecal/stool sample collection for future gut microbiome studies. Furthermore, methodological differences in the assessment of vitamin D “dose” [e.g., sun exposure, reported dietary, and nutritional supplement vitamin D intake, serum 25(OH)D] can lead to inconsistent results between studies.

Surprisingly, little is known about the direct effects of vitamin D on bacteria. This review identified a single study which demonstrated that vitamin D inhibited the growth of specific mycobacterial species *in vitro* (117). If this finding is corroborated, antimicrobial effects of vitamin D would be consistent with known immunoregulatory properties. If these findings are not corroborated, then it is likely that microbiota are mediated indirectly by vitamin D's immunologic properties [reviewed in (3)].

In contrast, there is data to support that bacteria actually influence vitamin D metabolism as some bacteria express enzymes involved in hydroxylation of steroids and thus are capable of processing and activating vitamin D in a manner similar to humans (118). Bacterial CYP105A1 (*Streptomyces griseolus*) converts vitamin D3 into 1,25(OH)2D3, in two independent hydroxylation reactions, representing the bacterial functional equivalent of the combined activity of vitamin D metabolic enzymes CYP2R1, CYP27A1, and CYP27B1 (119). Additional review of a microbial genome database for CYP27A1 and CYP27B1 revealed homologous protein from *Ruminococcus torques* (Phylum: Firmicutes) *Mycobacterium tuberculosis*, respectively (120). Capitalizing on these microbial enzymes, there is even a patent (U.S. Patent 5474923) for a process by which hydroxylated vitamin D derivatives are obtained by incubating vitamin D with *Nocardia, Streptomyces, Sphinogmonas*, and *Amycolata*. Additional studies are needed to understand the relationship between vitamin D and gut bacteria, and the role of bacteria in maintaining adequate vitamin D levels. Additionally, other factors responsible for modulating this relationship, such as FGF23 which decreases vitamin D in germ free mice, are important to investigate and understand how they influence this process (121).

### Vitamin D Supplementation and Changes in Autoimmune Disease Microbiome

Autoimmune disease and vitamin D deficiency are known co-morbidities, such that vitamin D supplementation for autoimmunity is a common practice. Thus, far we have discussed the importance of the microbiome in autoimmunity, as well as the ability for vitamin D to impact microbiome composition along the GI tract. However, not much is known about how vitamin D supplementation (or deficiency) impacts the microbiome of autoimmune patients. Only a few studies summarized below have addressed this question.

In a 4-week long vitamin D intervention for vitamin D deficient CD patients in remission, there were reduced bacterial taxa and changes in bacterial abundance following supplementation, without an effect in healthy controls (122). *Megasphaera* and *Lactobacillus* were enriched at week 4, but still comprised a relatively low abundance overall (122). A study of active and inactive UC patients found overall microbiota diversity to be unchanged following 8 weeks of vitamin D supplementation, but a significant increase in abundance of *Enterobacteriaceae* (phylum: Proteobacteria) in UC patients (123). Healthy control mice fed a high vitamin D diet (10,000 IU/kg) displayed reduced species diversity and were enriched with *Paludibacter* (phylum: Bacterioidetes) and *Sutterella* (phylum: Proteobacteria), the latter of which was also enriched in DSS colitis mice (124). Interestingly, DSS colitis mice fed this high vitamin D diet displayed a worsened colitis phenotype compared to moderate (2,280 IU/kg) or no vitamin D (0 IU/kg), suggesting that excessive vitamin D intake could promote a disease-exacerbating microbial community consistent with colitis (124).

Finally, a small study assessed fecal bacterial communities of 2 untreated and 5 glatiramer acetate-treated MS patients, and 8 healthy controls after vitamin D3 supplementation (79). Although there were only 2 untreated MS patients, these patients demonstrated an increase in *Faecalibacterium* (phylum: Firmicutes), *Akkermansia* (phylum: Verrucomicrobia), and *Coprococcus* (phylum: Firmicutes) following vitamin D supplementation compared to healthy controls and treated MS patients (79). Interestingly *Faecalibacterium* and *Akkermansia* have been reported in the IBD/colitis literature to be protective against disease (85, 125).

### Vitamin D Supports Intestinal and Immune Cell Defenses at the Gut-Immune Interface

The intestinal epithelium is in constant interaction with the external environment. Adequate barrier integrity and antimicrobial function at epithelial surfaces are critical in maintaining homeostasis and preventing invasion or overcolonization of particular microbial species. A healthy intestinal epithelium and intact mucus layer are critical to protect against invasion by pathogenic organisms, and vitamin D helps to maintain this barrier function.

Data supporting a role for vitamin D in maintaining tight junctions stem from studies of VDR^−/−^ mice that demonstrate susceptibility to invasive bacteria and LPS as measured by a reduction in transepithelial resistance. In contrast, vitamin D supplementation in the setting of functional VDR strengthens the epithelial barrier by reducing paracellular permeability of polarized epithelial cells (126–128). Multiple studies found that vitamin D3/VDR signaling modulates tight junction protein quantity and distribution. For example, there is reduced expression of ZO-1, occludin, and claudin-1 in DSS-treated Caco-2 cell culture that is at least partially rescued by the addition of 1,25(OH)2D3 (129), and SW480 cells treated with 1,25(OH)2D3 enhanced ZO-1, claudin-1, and E-cadherin protein expression (128). Similarly, tight junction mRNA transcripts and proteins were reduced in epithelial cell lines exposed to bacteria or LPS, and rescued with 1,25(OH)2D3 supporting a role for bacterial disruption of the barrier (127). In contrast, claudin-2, known to be a “leaky” tight junction protein, was found to be upregulated upon vitamin D3 supplementation (128) and downregulated in VDR−/− mice (130). As a “leaky” protein that allows movement of ions into the intestinal lumen, claudin-2 expression in the setting of functional vitamin D deficiency may contribute to colitis pathology. Furthermore, deletion of VDR from intestinal or colonic epithelial cells led to profound intestinal epithelial cell apoptosis (131).

Vitamin D upregulates antimicrobial peptide mRNA and protein expression including cathelicidin (132), defensins (133), and lysozyme (112) *in vitro*, and *Ang4 in vivo* (134). Antimicrobial peptides, primarily secreted by Paneth cells in the gut, are important mediators of microbiome composition as shown by *in vivo* studies demonstrating, for example, increased bacterial translocation following Paneth cell ablation and increased susceptibility to colitis or pathogen infection [reviewed by (135)]. Cathelicidins are secreted at surfaces interacting with the external environment where they are capable of forming transmembrane pores in the bacterial cell wall and also have antiviral and antifungal properties [reviewed by (136)]. Defensins are secreted by epithelial cells, Paneth cells, and immune cells, and are important components of the innate immune response in the gut. Loss of VDR expression by intestinal epithelial cells led to abnormal Paneth cells, reduced lysozyme mRNA expression, impaired autophagy, and increases in *E. coli* and *B. fragilis* (112). Finally, vitamin D deficiency has been associated with reduced colonic expression of Ang4 and a 50-fold increase in colonic bacterial infiltration in mice (134).

## How May Vitamin D Deficiency Affect Intestinal Bacteria and Orchestrate Autoimmunity?

**Figure 2:**
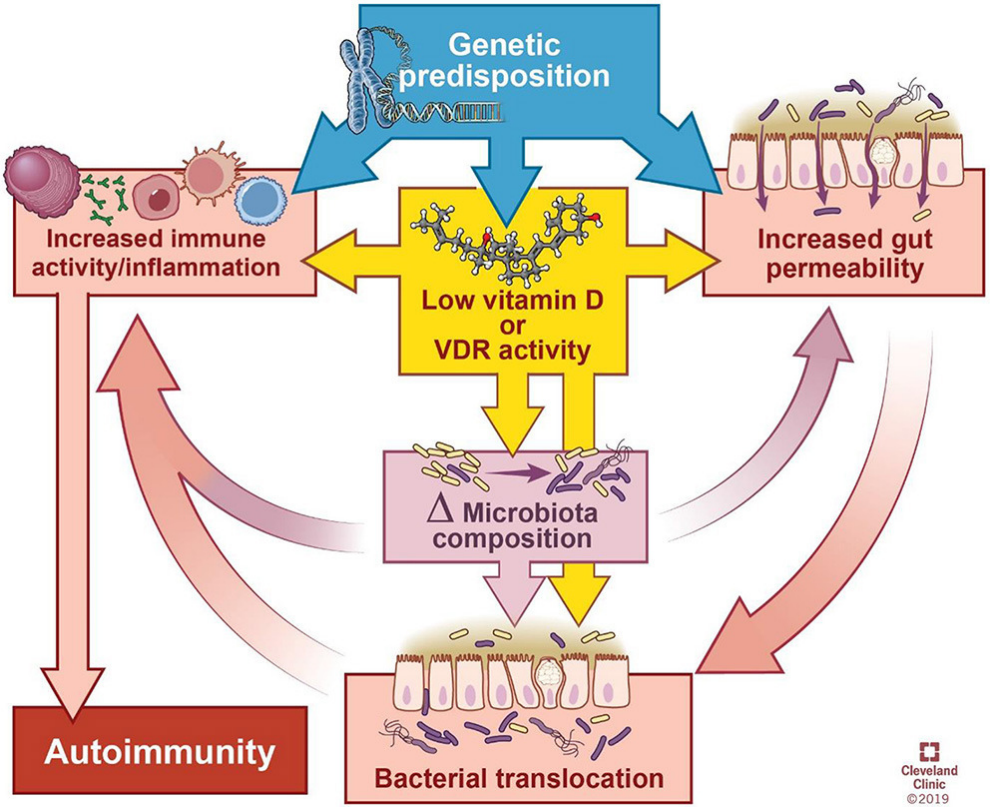
Model of the interactions between genetics, gut integrity, microbiome, and vitamin D deficiency. Genetic predisposition can influence vitamin D activity, integrity of the gut barrier, and basal level of immune activation. Low vitamin D increases the permeability of the gut barrier and heightens immune activity. Furthermore, low vitamin D and permeability of the gut alter microbial composition and the ability of microbes to translocate across the intestinal epithelium, leading to interaction with the host immune system. Ultimately, immune system activation contributes to autoimmunity. Δ = change. Illustration by David Schumick, BS, CMI. Reprinted with the permission of the Cleveland Clinic Center for Medical Art & Photography © 2019. All rights reserved.

Based on the evidence presented above, we suggest that vitamin D deficiency may affect the microbiome and the immune system hereby contributing to autoimmune disease (Figure 2) as follows:

1. Vitamin D deficiency or supplementation changes the microbiome, and manipulation of bacterial abundance or composition impacts disease manifestation.
2. Lack of vitamin D signaling due to dietary deficiency or genetic impairment of VDR expression/activity can impair physical and functional barrier integrity (Figure 1). This allows bacteria to interact with the host leading to stimulation or inhibition of immune responses.
3. Our natural, innate immunologic defenses may be compromised in the setting of vitamin D deficiency.

As evidenced by *in vitro* and *in vivo* studies reviewed elsewhere (3), vitamin D acts directly on immune cells to promote an anti-inflammatory state, and the balance between proinflammatory and anti-inflammatory activity is disrupted in vitamin D deficiency in favor of the former. Despite the numerous ways in which vitamin D can affect the immune system, vitamin D deficiency alone is insufficient to initiate autoimmunity. However, through its effects on bacterial communities, epithelial integrity, or immune function, vitamin D has the capacity to exacerbate other predispositions, such as genetic polymorphisms (e.g., in VDR, metabolic enzymes, intestinal barrier function, immune function), dietary and environmental factors. As depicted in Figure 2, the cyclic influences between these factors obscure cause and effect relationships. While a genetic predisposition(s) is required, it is possibly the simultaneous convergence of non-genetic factors upon a genetically susceptible individual that results in autoimmunity.

## Conclusion

In this review, we discussed an abundance of evidence that demonstrates the importance of the microbiome; however, establishing temporality of dysbiosis has remained difficult. As with many diseases, animal studies must be utilized to understand causation. Future clinical longitudinal microbiome studies of individuals with first-degree relatives with autoimmune disease would be beneficial in this effort. It is possible that microbial changes may precede and initiate disease in one entity, yet prove to be passive responses to disease in other entities.

Specific patterns of dysbiosis are now being associated with autoimmune diseases. This is exciting as it unveils new targets that can be leveraged for therapeutic potential. However, much work remains in order to fully understand this complex system. Single pathogen associations such as *Faecalibacterium prausnitzii* and IBD are just the beginning as we begin to consider not only the resident bacterial community composition, but also bacterial networks based on function, metabolites, etc. and how these networks influence disease. It is evident that the immune system and microbiome are interconnected, and that vitamin D is a critical intermediary player in this dynamic. Thus, there is a need to expand our understanding of the implications of vitamin D deficiency and supplementation on bacterial communities in both states of health and autoimmunity. The studies on vitamin D and gut health are, understandably, focused on IBD. However, if the reciprocal influences of vitamin D, gut barrier function, microbiome, and immune responses are true, then there is much to be gained from pursuit of these studies in all autoimmune diseases.

## Author Contributions

EY was responsible for the primary review of literature, consolidation of information, and writing. TJ was responsible for oversight, significant guidance/direction, and critical review/editing.

### Conflict of Interest

The authors declare that the research was conducted in the absence of any commercial or financial relationships that could be construed as a potential conflict of interest.
